# The Patients' Christmas Day

**Published:** 1911-12-30

**Authors:** 


### THE PATIENTS' CHRISTMAS DAY.

### SPLENDID RECORD FROM ALL PARTS OF THE COUNTRY.

This year, as usual, the newspapers contain rather perfunctory notices of the means by which hospital workers try to make those in the wards forget their ill-health at Christmas-time. The fact that amusements and decorations take place in hospitals at this season is well known, but who inspires them, who organises them, the complicated arrangements they necessitate, the names of the voluntary workers, these are unsuspected or unknown. Yet in these details will be found the spirit of hospital Christmas, and the secret of the patients' enjoyment, and therefore this year we have decided to publish brief accounts of what actually took place in some of the most characteristic hospitals of London and of the provinces. These miniature pictures have the charm of accuracy and detail, and bring out the spirit that has just gone to the making of the patients' Christmas Day in the wards.

### A Farce by the Matron at the Metropolitan.

Christmas Day was ushered in for the 123 patients then in the Metropolitan Hospital by nurses walking round the wards carrying lamps and singing carols. As the hospital has been so recently redecorated, no evergreens were used for Christmas decoration, but the wards were made festive by pretty lamp shades and a plentiful supply of flowers. After breakfast Father Christmas appeared, his character being taken by one of the resident staff, who was accompanied by a clown, a policeman, and others. Every patient thereupon received a parcel, those for the men containing a shirt, necktie, and a pair of socks, and those for the women a petticoat or shawl. To the children Father Christmas brought toys, many of which had been given by the kind Editor of *Truth*, and the wards began to resound with the blowing of trumpets and those other noises that are inseparable from a children's Christmas morning. All patients who are able to take turkey and plum pudding are carved for by the doctors and sisters in the wards. The Christmas pudding was carried flaming round the ward amid cheers like those of the little Cratchets in Dickens.

All the patients are allowed to smoke on Christmas Day; the men's tobacco, cigarettes, and pipes were kindly provided by Mr. J. Dunnington Jefferson, Mr. A. Lister Harrison, besides the following tobacconist firms: Messrs. W. D. and H. O. Wills, Ltd., ever to be connected with the late Lord Winterstoke and the Bristol hospitals; Lambert and Butler, and Adolph Frankau and Co. As the women and children do not smoke, sweets were provided by the kindness of Messrs. Fry, Cadbury, and Maynard. In the afternoon visitors were allowed in, and every patient could keep one friend to tea and to spend the evening. It was, in fact, the patients' "at home" day.

After tea, with fruit and cakes and plenty of crackers—given, these last, by the Christmas Entertainment Fund—the evening performance began. This included a variety show by the resident medical staff and the nurses, "The Area Belle," a farce, "Mechanical Jane," by the matron and two sisters, the fun of which cannot be conveyed to those who missed it; and a nurses' troupe and waxworks. The Misses Cresswall also played with violin and piano. These performances are given in two of the surgical wards, and all medical patients that are able to be moved are taken to enjoy the fun.

This was not the last of it, however, for on Tuesday last Mr. G. Nathan gave a concert, while the day and night nurses' dinner party was on Wednesday and Thursday. On Saturday the out-patient children are to be entertained to tea; and on Tuesday, January 2, the Christmas tree begins, when the ward children participate.

### Keeping Christmas Eve at Cardiff.

It has long been the custom at King Edward VII.'s Hospital to commence the Christmas festivities on Christmas Eve, when the great tree in the children's ward is lighted up and the presents hung on its branches are distributed by Father Christmas and his staff. Saturday, the 23rd, was this year recognised as Christmas Eve for this purpose, and at five o'clock in the afternoon the children's hearts beat high and their voices rang loud when the electric light was switched on, revealing the great tree laden with presents for every inmate of the hospital, its branches twinkling with miniature electric lamps, and streamers, also electrically lit, leading in all directions from the tree to the ceiling of the ward. Then the Lord Mayor, Alderman J. W. Courtis, mounted a ladder and apologised that Father Christmas had not arrived, as his motor had broken down and his beard was lost in the rain. The Lord Mayor cut off the first present and several other presents after that, with the expert assistance of Dr. Herbert Vachell, the chairman of the Medical Board, Dr. Maclean, Dr. Paterson, Dr. A. Taylor, Dr. Cook, and Mr. William Martin, while the Lady Mayoress and the Matron supervised the distribution of the presents to the various wards. Much merriment was caused by the gifts for the medical staff, which as a general rule wittily hit off some personal characteristic. In the course of half an hour Father Christmas, who was admirably impersonated by one of the house surgeons, Dr. Armstrong, made his belated appearance, and the fun became fast and furious, the children shrieking with delight, while their elders entered whole-heartedly into the true expression of a "Merry Christmas." One little girl, from Yuysbwl, caused much good-natured amusement by identifying Father Christmas as Mr. Lloyd George, and she will return to her home in the hills with grateful recollections of the Chancellor. Shortly after six o'clock the company repaired to the Insole Ward, where just the right sort of concert was provided by "The Pom Poms" Troupe of Pierrots, under the leadership of Mr. J. Ewart. In the wards generally the decorations were not so elaborate as usual, although a special feature was made of the children's ward. On the whole it was considered that the simplicity of the decorations conduced to the comfort of the patients, and every ward was well lighted and cheerful and brightened by happy faces. On the Sunday the services at the hospital were well attended, and the hymns chosen were, of course, distinctive of the sacred season.

The morning of Christmas was a time of expectancy, and during the forenoon many members of the staff visited their patients and wished them the compliments of the season, and shortly after noon the Lord Mayor and Lady Mayoress visited each ward and extended a hearty greeting to the patients, which was much appreciated. Dinner was probably the great event of the day—the *pièce de résistance*—and a happy note was struck by the appearance of the nurses, who came from their own dinner to wait upon the patients, adorned in the fantastic caps and favours from their Christmas crackers. The patients hugely enjoyed their dinner, which consisted of turkey and ham and plum pudding. Members of the honorary and resident staff did the carving in each ward, and after the meal, when crackers and fruit were distributed, there was an interchange of fun and friendliness which was as good as a tonic. In the afternoon the patients' friends came to see them, while gramophones were played at frequent intervals; and after tea, of which a specially decorated Christmas cake was a feature, concerts, provided by kind friends of the hospital, were kept going in one or other of the wards up to 8.30 p.m.

Throughout the remainder of the week, on succeeding nights, each ward will have its special concert, and these are looked forward to with eager anticipation, and will gently break the gradual return to the ordinary routine which will set in with the New Year. Dr. Shepherd, the resident medical officer, and the house surgeons, Dr. Harries, Dr. Armstrong, Dr. Rocyn Jones, and Dr. Inglis, threw themselves whole-heartedly into the amusements of the patients, and the entire arrangements reflect the greatest credit on the devoted preparation of the matron, Miss E. A. M. Wilson, aided by the assistant matron, Miss Hutchinson. The only drawback arose from the indisposition of the Chairman of the House Committee, Colonel Bruce Vaughan, who was prevented from extending his greetings to the patients in person, but the Colonel did not fail to send his Christmas message to the hospital, which, in accordance with his custom, assumed a tangible and much appreciated form. It should be mentioned that the friends of the patients were free to visit them on Saturday, Sunday, Monday, and Tuesday afternoons, and this was probably the most human and most enjoyable part of the Christmastide.

### At the Manchester Royal Infirmary.

Christmas at the Manchester Royal Infirmary commenced with gifts for every patient, provided from a special fund collected from the members of the Board of Management, the medical staff, and other friends by Miss Sparshott, the lady superintendent of nurses, after which there was Communion service in the chapel, followed by a short service at 9.45. The day was then given up to amusement. This began with a great gathering of children in the Out-patient Hall. All of them were out-patients, and many in splints and bandages, who had assembled in accordance with time-honoured custom to receive presents of dolls and other toys, buns, oranges, and apples. There was a huge gaily decorated Christmas-tree and hundreds of presents, and it was delightful to see the eagerness exhibited by the children and their mothers as the little ones waited to receive their presents from the hands of Sisters Hutchinson and Hill, the out-patient and casualty sisters (whose pleasure it is each year to collect the presents or the wherewithal to obtain them from the members of the staff, the students, and many other contributors), and their helpers. This year the proceedings were enlivened by an excellent gramophone entertainment, kindly provided by a member of the Board of Management. The necessary ward work had been completed by the time the last small out-patient departed, and there was then a general inspection of the wards by the chairman of the Infirmary Committee, Mr. R. P. Goldschmidt, the General Superintendent (Mr. Carnt), the Lady Superintendent (Miss Sparshott), and other friends. The decorations, which were chiefly floral, were much admired. The various dining-rooms for the staff were also inspected, the tables being arranged with much taste. Smoking was permitted in the men's wards after dinner. Concert parties, which included several of the resident medical staff and students disguised in fantastic dress, Father Christmas, Harry Lauder, Golliwogs, &c., among them being particularly conspicuous, proceeded from ward to ward, gramophones being also utilised to fill up gaps between the visits of the entertainers. Special teas were provided, the resident officers and nurses having tea, and after the labours of the day were over, supper in their own units. At seven o'clock the servants' Christmas party began, and they enjoyed themselves thoroughly until eleven o'clock, a very amusing one-act play by six of their number occupying an important position in the evening's programme. Other amusements and entertainments will take place nearly every day during the week.

### How Christmas was Kept at Birmingham.

On Christmas Eve we began our celebrations, for before going on duty at 9 P.M. the night nursing staff sang carols round the wards and corridors. On Christmas morning at the General Hospital, Birmingham, according to custom, each patient found on his bedside locker a parcel containing clothes, together with a Christmas card. In the chapel Holy Communion was celebrated soon after six o'clock and 8 A.M., and there was a morning service, with carols, at 11. Then the event of the patients' dinner at 12.15 took place. It consisted of roast beef and plum pudding, or chicken and custard, with an apple and orange to follow. Pipes and tobacco or cigarettes were given to all the men patients who smoked, and they were allowed to smoke in the wards during the afternoon and evening. At three, Father Christmas, accompanied by a troupe of the resident doctors in fancy dress, visited all the wards and distributed toys to the children. Soon after this a concert was held in one of the accident wards. All the performers were members of the resident and nursing staffs. Boxing Day was the day of the ward maids, and the servants' dinner was held at 2 P.M., the carvers and waiters being the resident doctors. Here indeed it might be said that the octave is kept, for on Friday night an entertainment was given in the Out-patient Hall to those patients able to be moved down by ambulance or wheel-chair, as well as those who can walk, and to the servants. This entertainment consisted of plays given entirely by the resident medical and surgical officers. On Wednesday at the Jaffray Branch Hospital a tree and entertainment were given in the afternoon, and on Thursday an entertainment was given in the Out-patient Hall by the nursing staff, consisting of plays, choruses, and songs. On Friday the three trees in the two children's wards and the Burn Ward, which were loaded on Christmas Eve, are to be stripped, and a ward concert held. Here, by the way, heavy decoration of the wards is not encouraged, but all the wards are brightened by the sisters and nurses. Here, too, as is now fairly common for the purposes of those entertainments, a fund is raised by the house governor from members of the board, staff, and other friends of the hospital, so that no part of the expenditure falls on the hospital funds. But a more informal feature is that a small gift is distributed to each member of the staff, from the highest to lowest, on Christmas morning, and tips are given to the porters and servants who have the extra work during Christmas week.

### The Bishop's Visit to Liverpool Infirmary.

An innovation in our keeping of Christmas Day at the Royal Infirmary, Liverpool, was due to the kindness of the Bishop of Liverpool, who was present at the early celebration. The wards were all decorated with great secrecy beforehand but hangings are not allowed, and the decorations consisted of flowers, both cut and growing, with the initials of the honorary staff. One of the wards was, perhaps, the most effective, with groups of beautiful scarlet poinsettas. The electric-light shades matched the floral decorations in all the wards. In another ward the Sister had a group of three sets of initials, with "Centenary, 1811-1911," this year completing a hundred years the Bickersteths have been members of the honorary staff, being the completion of three generations. The patients throughout the day—even before they had their gifts—appeared unable to express their great enjoyment. Tobacco and pipes were given to all the men, and they were allowed to smoke. The nurses sang carols in the corridors at 5.30 in the morning. The patients' dinner was at 12 noon, and consisted of turkeys, plum puddings, and jellies, with dessert and crackers, when the members of the honorary staff and committee visited the wards and carved. An hour later the nurses were at dinner, the sisters taking charge of the wards. On Thursday the Christmas tree is to be unveiled in the new out-patients' hall. The patients' gifts are also to be distributed then, the Lord Mayor and Lady Mayoress (Lord and Lady Derby) having promised to come.

### Elaborate Entertainments at Leeds.

Christmas Day at the Leeds General Infirmary was one of brightness and gaiety. All the wards were gaily decorated, some with evergreens and holly, others with more ambitious efforts, made to represent Japanese gardens, resplendent with bowers of delicate apple-blossom. One ward which last year illustrated "Blue Bird" on this occasion became the "Cracker" ward, the decorations representing crackers of all sizes, from the miniature of one inch to huge ones twelve feet long. The Doctor, who dexterously wielded the "scalpel" to the sirloin of beef in the ward, appeared to be an automatic cracker standing seven feet high. Santa Claus visited the institution and left a seasonable and useful memento with each patient. After dinner, fruit, chocolates, and tobacco were the order of the day. It made one almost envious to see the delight with which some of the old men—and younger ones, too—pulled at their pipes after an enforced abstinence of some days or weeks.

During the evening the Lady Superintendent, leading a choir of nurses, visited each ward and sang carols, much to the pleasure of the patients.

Wednesday and Thursday evenings as many patients —about 300—as could be removed to the Out-patient Hall were charmed with the entertainments provided by the resident medical staff and night nurses, the performances being interspersed with topical songs, calling attention to grievances, imaginary and otherwise, and to eccentricities of members of the honorary staff and the authorities of the Infirmary. In the children's wards were Christmas trees, the boughs of which were laden with dolls, steam engines, and other up-to-date toys; numbers of tiny electric lights illumined the trees each evening till Thursday, when Father Christmas, with great solemnity and many stale chestnuts, disrobed the trees and distributed the good things to the delighted little invalids.

### The London Fever Hospital.

At a fever hospital there must of necessity be a good deal of restriction in the matter of Christmas entertainments, but at the London Fever Hospital in Islington the Matron, Miss Fleming, and her assistants managed to give the patients a real good time on Christmas Day. We are informed that Dr. Lytton Maitland, the Medical Superintendent, disguised as Santa Claus in the flesh made his appearance in the wards with enormous sacks, borne by his faithful henchman, the clown, and distributed presents to everyone. As usual, there was a large proportion of children in the wards and, fortunately most of them were able to participate in the games and music subsequently provided. All the wards were cheerfully decorated, but the diphtheria block scored rather heavily with its electrical illuminations in the entrance lobby! A stage had been erected in an empty ward, and a staff entertainment was given on Wednesday evening; as many of the patients as can be moved will have the opportunity of seeing a repetition of this on Friday. A most enjoyable programme was submitted, and the amount of unsuspected local talent revealed surprised everyone. The usual staff dinners took place on Tuesday and Wednesday, but the annual dance and the sisters' dinner will be held on January 17.

### The "Tree" at the Middlesex.

Christmas at the Middlesex Hospital was truly magnificently celebrated, and there was no lack of entertainment for the patients and their friends who attended in crowds. The large and beautifully arranged Christmas-tree in the Princess May Ward was a great centre of attraction, while of the ward decorations, those in Broderip Ward were perhaps the most effective. In the matter of concerts, etc., the patients were well served—Mr. Mackenzie, one of the house surgeons, having a large share in the highly successful arrangements. Other entertainments were given during the week, and the Matron and the Resident Medical Officer are to be congratulated on the splendid result of their labours.

### A Success for Local Talent at St. Thomas's.

The Christmas festivities at St. Thomas's Hospital extended throughout the week, and the patients were treated royally to innumerable shows. Local talent rose splendidly to the occasion, and provided an excellent band of Pierrots, as well as many sporadic entertainers. On Christmas Day the wards were invaded by crowds of friends, whose appreciative exclamations at the sight of the decorations must have been pleasant hearing to the workers responsible. Out of so many wards it is impossible to say which was the most beautiful to look upon, but of those seen by our representative, the "Elizabeth" ward was one of the most effectively decorated. The carol-singing by a choir of nurses and students was voted this year to be better than ever.

### The Pierrots at St. Bartholomew's.

It must have required no small amount of organisation to have so successfully entertained the large number of patients in St. Bartholomew's Hospital on Christmas Day. In each of the numerous wards were "turns" given by troupes of pierrots, singers, and reciters in fantastic garb, and it speaks volumes for the institution that so much talent was available from among the students, nurses, staff, and their friends. All the wards were most tastefully decorated, and those familiar with their everyday aspect might well have rubbed their eyes on surveying the charming effects produced by the skilful work of the sisters and their helpers. On Boxing Day a concert was given in the out-patients' hall for those patients able to leave their beds.

### The Prettiest Ward at Charing Cross.

At Charing Cross Hospital there was no lack of the true Christmas spirit—every ward was beautifully decorated, and the sisters were busily engaged in dispensing tea and cakes to the numerous visitors. If we had to award the palm to one particular ward we should have to single out the Golding Ward as the most pleasing to the eye, though it must be confessed that two others ran it very close. During the afternoon, concerts, recitations, etc., were given by obliging friends, among whom were to be noted Mr. James Welch, of "knightly" fame, and Mr. Collett. On Christmas Eve carols were sung round the wards by the nurses and students. The grand concert and entertainment for the patients will take place on January 4 in the out-patients' hall, and the Resident Medical Officer, Mr. D. Powell, is to be congratulated on the splendid result of his hard work in this direction. The annual dinner and dance, given by the resident medical officers to the sisters and matron, took place on Boxing Day.

### The Minstrels at Westminster.

At Westminster Hospital the patients were enlivened by wandering minstrels in the morning of Christmas Day, and by a grand concert in the Board Room in the afternoon. The wards were gaily illuminated, but no very ambitious decorative efforts were undertaken. During the week many concerts and other entertainments were given in the wards.

### The "Drummond" Ward at St. George's.

Christmas Day at St. George's Hospital was comparatively quiet, as the great day of festivity for the patients and their friends was Thursday, when all sorts of good things were sent out for their benefit and entertainments galore were in full swing. But Monday was not without its Christmas air, for the carol-singing was a great success this year. All the wards looked most charming with their tasteful decorations, but at the risk of making invidious distinction we are inclined to think that "Drummond" was the most successful.

### An Infirmary Christmas.

The festivities were in full swing at the Camberwell Infirmary when our representative arrived at 1.30 P.M. on Christmas Day. The nursing staff were conspicuous by their absence at their own Christmas dinner. Dr. Keats, the medical superintendent, very kindly did the honours and explained the decorations in the wards, which were very tasteful and excellently done. Each ward was left to provide its own amusements, and this they did with the utmost vigour. For instance, in the Men's E 1, those who were able to be up were seated round the blazing fire at the end of the ward telling stories and occasionally bursting into song, while passing through the children's wards one saw nurses romping with those who were up, to the great amusement of those who were confined to their beds. An especial compliment should be paid to those who were responsible for the decorating of the Children's Ward F, for this was, in its own fashion, the prettiest of the wards, which, however, had an added charm from their large number of bonnie children, that certainly enhanced the effect.

### THE MORAL OF IT ALL.

THE above accounts have a special interest from the fact that all unconsciously they reveal the attitude of each hospital towards, not merely the patient, but the public. Hospitals, like actors, must keep in the public eye, and THE HOSPITAL threw open its columns this week to such accounts as might arrive from all quarters. The result shows that there is still something to be done before the publicity department of every hospital takes full advantage of the opportunities afforded by its generosity to patients at Christmas time. The opportunity in some cases, it will be seen, has been magnificently recognised.

